# An energy optimization study of the electric arc furnace from the steelmaking process with hot metal charging

**DOI:** 10.1016/j.heliyon.2022.e11448

**Published:** 2022-11-07

**Authors:** Anton Irawan, Teguh Kurniawan, Hafid Alwan, Zaenal Arifin Muslim, Hidayathul Akhmal, Mochamad Adha Firdaus, Yazid Bindar

**Affiliations:** a Chemical Engineering Department, Universitas Sultan Ageng Tirtayasa, Serang 42122, Indonesia; b RT Aksiatama Foundation, Cilegon 42443, Indonesia; c PT Krakatau Steel, Cilegon, Indonesia; d Chemical Engineering Department, Institut Teknologi Bandung, Bandung 40116, Indonesia

**Keywords:** Electric arc furnace, Hot metal, Dynamic simulation, CFD, Combustion

## Abstract

A study of energy optimization derived from simulation software has been done for the high-intensity energy of an Electric Arc Furnace (EAF), hot metal charging within the cooler duct dedusting system. The present work aims to develop a dynamic model of the EAF operation based on mass and energy balances integrated with simulation of the dedusting system with hot metal charging system using MATLAB® and Computational Fluid Dynamics (CFD). The effect of various percentages of hot metal charging on EAF performance and the dedusting system was simulated and validated from the real EAF data plant from one of the steel manufacturer companies in Indonesia. Three cases for the EAF with various hot metal (HM), sponge iron, and scrap iron charging compositions and four cases for the EAF with post-combustion CO have been developed. Careful observation shows that the electric arc power consumption can be reduced down to 72.9 MWh from 87.4 MWh (ca. 16% more efficient) while at the same time increasing the HM charging temperature at the endpoint of the duct dedusting system up to 900 °C from 540 °C (app. 65% higher). Additionally, advanced simulation of an EAF with post-combustion CO shows that power consumption can be decreased to 59.9 MWh (ca. 30% more efficient).

## 1 Introduction

The simplest technology of iron making is produced by the smelting process [1]. This iron then could be processed further to make steel by several techniques. There are two commercial routes for steelmaking plants in the modern era, i.e., basic oxygen steelmaking and electric arc furnace. An electric arc furnace (EAF) method utilizes a furnace coupled with an electric arc to supply heat for heating and melting scrap. The EAF is the most common process technology for scrap recycling to reuse waste components into more beneficiary materials. Scrap components are accounted for 75% of the EAF feedstock, while direct reduced iron (DRI) and hot briquetted iron (HBI) cover ca. 15%, and the rests are pig iron and hot metal. The EAF technology is also used for recycling internal wastes such as the EAF dust, refractory materials, and slags [2].

The iron industry is an industry that operates by intense energy to run its process. Therefore, increasing the energy cost requires various methods to reduce energy consumption in the iron and steel industry, including EAF [3]. The need to seek efficient EAF operations which offer low cost and are environmentally friendly has been attracting attention for decades. One of the operation methods is by combining recycled scrap with alternative feedstock, which can also come from the internal steelmaking process, such as DRI, HBI, hot-metal, and pig iron. By default, cold scrap and sponge iron are the main materials of EAF feed. Besides scrap and sponges, hot metal is an attractive alternative material. A low-cost matter with high energy content produced from blast furnaces. Mixing traditional EAF feed with hot metal offers advantages such as reducing power consumption, low carbon emission, and reduced lime consumption [4], [5], [6].

Generally, hot metal is a virgin metal that contains lower traces of impurities than scrap. Accumulation of impurities brings difficulty in steel manufacturing, such as hot shortness during rolling and temper brittleness [7]. The open literature reported that hot metal charging in industrial EAF capacity 50 and 130 ton/h successfully reduced power consumption [6][8]. Modeling and simulation approaches based on the first principle of mass and energy conservation are effective tools for analyzing the EAF system. A static mass and energy model of EAF combined with a MgO saturation slag model was reported by Arzeypema et al. [9]. The model is suitable for selecting raw materials, predicting energy consumption, and calculating element content in melt and slag properties. Also, the dynamic model for the closed-loop control system EAF can be based on the first principle model of mass and energy conservation [10]. Logar et al. further developed the dynamic model to improve thermo-chemical reactions [11][12]. Model development of EAF was also reported by Meier et al. [13], which included gas components H_2_, H_2_O, and CH_4_ in the model.

Another aspect that can be optimized for model development in the EAF is the dedusting section. A dedusting system is required in EAF operation to pre-treats high-temperature off-gas and removes dust before releasing it into the atmosphere. Normally, a dedusting system consists of a dust dropbox, water-cooled duct, uncooled duct, dust filter bag, and stack. The feeds and operating conditions of the EAF are significantly affected by the dedusting system performance. In addition, post-combustion CO and H_2_ with air intrusion through slip gap occur in the water-cooled duct section releasing heat in the water-cooled duct area. Kolar et al. developed a Sankey diagram of the EAF dedusting system to analyze steam generation potential in the water-cooled duct area of the dedusting system [14]. The cogeneration plant was analyzed to utilize heat from the dedusting system, which potentially reduced the costs of power, steam, and cooling water by 1.5, 32, and 29%, respectively. [15].

The more sophisticated computational fluid dynamic (CFD) model is gaining interest as it offers more reliable aspects, including transparency, comprehensive, and safer for EAF simulation [16]. However, the CFD simulation particularly in the arc furnace is complicated and tedious. Hence, conventional dynamic mathematics model of the arc furnace section is more practical than the CFD approach. The ordinary differential equations raised could be easily solved by using MATLAB. The CFD simulation in other sections of EAF such as dedusting section could be less complex and more beneficial to be applied. To the best of our knowledge, modeling, and simulation of integrated EAF with the dedusting system are rarely reported if it is none. The present work aims to develop a dynamic model of the EAF operation based on mass and energy balances integrated with CFD simulation of the dedusting system with hot metal charging. The effect of various percentages of hot metal charging on EAF performance and the dedusting system was simulated and validated from the real EAF data plant from one of the steel manufacturer companies in Indonesia. The following sections present the model development in EAF and dedusting system, model validation, simulation results of an existing condition, effect of hot metal charging, effect of oxygen flow rate, and post-combustion CO in the arc furnace.

## 2 Model development

### 2.1 Model development in EAF

**Figure 1:**
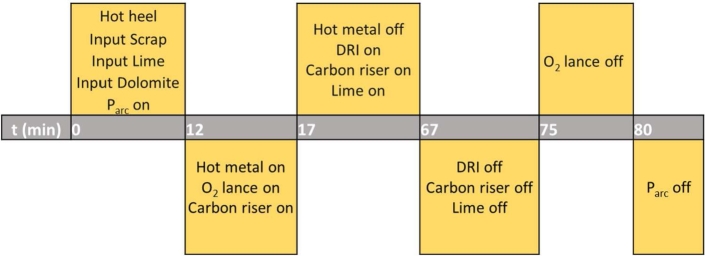
Timeline for simulation of EAF with hot metal charging based on daily EAF operation in PT Krakatau Steel.

Table 1 shows the characteristics parameter of the EAF used in this study. The timeline for the simulation of EAF with hot metal charging is presented in Fig. 1. Hot heel, which consisted of 99% metal, remained in the furnace to help preheat and accelerate scrap melting. The scrap was charged into the furnace, followed by lime and dolomite at the beginning of the batch process. Electric power was on to melt the scrap. After 12 min, hot metal was loaded, and the oxygen lance turned on. Five minutes later, the hot metal was off. DRI, carbon riser and lime were loaded. At 67 min, DRI, carbon riser, and lime input were stopped while oxygen remained blowing into the furnace. At 75 min, oxygen blowing was turned off. Finally, at 80 min, electric power was turned off.

**Table 1:** EAF characteristic data plant of present work.

Parameter (units)	Value
Capacity (Ton)	130
Shell Diameter (mm)	6750
Transformer (MVA)	93
Electrode - Polycrystalline Diamond (PCD) (mm)	1250
Sect. Volt (Max)	850
Total Annual Production (Ton/year)	960,000
Electrode diameter (mm)	610

The model was adapted from [10] with several modifications. Fourteen unknown variables with fourteen ordinary differential equations to be solved using MATLAB: T_ssc_, T_lsc_, m_ssc_, m_lsc_, m_lsl_, m_ssl_, m_CO_, m_CO2_, m_N2_, m_Si_, m_Fe_, m_FeO_, m_C_, m_SiO2_. Several assumptions have been considered to simplify the equations.

a. The liquid phase consists of iron and slag is at the same temperature. The gas-phase is assumed the same temperature with the liquid phase because the gas-phase is small compared to the liquid phase.

b. The solid phase temperature is at the same temperature as the solid scrap.

c. Heat from the arcs is all transferred to the liquid metal.

d. The gas-phase elements considered in the EAF models are CO, CO_2_, N_2_, and O_2_. Hydrogen is not included in the model as the content is small.

e. The iron oxide (FeO) and silica (SiO_2_) that form in the EAF are immediately dissolved in the molten slag.

f. The iron (Fe) in the DRI melts instantaneously when introduced into the EAF. The latent heat required to melt the iron was calculated in the model.

g. The impurities in the scrap are assumed not reacted except carbon.

The mathematics models for calculating solid scrap and liquid scrap temperature simulation are formulated based on the energy balance. The heat transferred from the liquid metal in the EAF to the scrap and the solid slag. The amount of heat transferred is linearly proportional to the temperature difference between the liquid metal and the solid scrap. Temperature of liquid slag is assumed the same with the temperature of liquid scrap to simplify the calculation. The rate of change for the solid scrap and solid slag temperature is defined as the heat transferred divided by the heat capacity of solid scrap and slag (Equation (1)).

(1) $$\frac{dT_{ssc}}{dt}=\frac{\left(k_{Fe}\alpha_{ssc}m_{ssc}\left(T_{lsc}-T_{ssc}\right)+k_{ssl}\alpha_{ssl}m_{ssl}\left(T_{lsl}-T_{ssl}\right)\right)\left(1-\frac{T_{ssl}}{T_{m}}\right)}{\left(\frac{m_{ssc}}{M_{Fe}}c_{p,ssc}+\frac{m_{ssl}}{M_{sl}}c_{p,ssl}\right)}$$

The rate of change for the liquid scrap temperature is function of heat input-output (${\overset{˙}{Q}}_{t}$), arc power ($P_{arc}$) and heat losses ($Q_{loss}$) as presented in Equation (2). ${\overset{˙}{Q}}_{t}$ is contributed by the chemical reactions, heat capacities, and latent heat. $P_{arc}$ is the electric arc power. The heat loss is proportional to the temperature difference between the EAF and the ambient temperature $Q_{loss}=k_{vt}\left(T_{lsc}-T_{air}\right)$. The liquid phase temperature rate is the total energy divided by the heat capacity of all the fluids as follows (Equation (2)).

(2) $$\;\frac{dT_{lsc}}{dt}=\frac{{\overset{˙}{Q}}_{t}+P_{arc}-Q_{loss}}{\left(\frac{m_{ssc}}{M_{Fe}}c_{p,lsc}+\frac{m_{C}}{M_{C}}c_{p,C}+\frac{m_{Si}}{M_{Si}}c_{p,Si}+\frac{m_{lsl}}{M_{sl}}c_{p,lsl}+\frac{m_{FeO}}{M_{FeO}}c_{p,FeO}+\frac{m_{SiO_{2}}}{M_{SiO_{2}}}c_{p,SiO_{2}}\right)}$$

The mathematics models for calculating the rate of change for the solid scrap mass is derived from the energy balance, which is proportional to the heat transfer rate from the liquid to the solid phase (Equation (3)). The driving force is the temperature difference between solid and liquid temperature.

(3) $$\frac{dm_{ssc}}{dt}=\frac{-M_{Fe}K_{area1}K_{therm1}m_{ssc}\left(T_{lsc}-T_{ssc}\right)}{\left(\lambda_{ssc}+c_{p,Fe}(T_{ssc}-T_{lsc})\right)}\frac{T_{ssc}}{T_{lsc}}$$

The liquid metal mass ($m_{lsc}$) is determined by several factors, i.e., the melt rate of the steel scrap, the rate of oxidation of Fe by O_2_ to FeO, the reduction of FeO to Fe by graphite injection into the slag and by dissolved carbon and silicon in the steel melt, DRI and hot metal mass rate input. The rate of change of liquid scrap is given by Equation (4) as follows.

(4) $$\frac{dm_{lsc}}{dt}=\frac{M_{Fe}K_{area1}K_{therm1}m_{ssc}\left(T_{lsc}-T_{ssc}\right)}{\left(\lambda_{ssc}+c_{p,Fe}(T_{ssc}-T_{lsc})\right)}\frac{T_{ssc}}{T_{lsc}}\;\;+\frac{m_{FeO}k_{gr}M_{Fe}C_{inj}}{M_{C}\left(m_{lsl}+m_{FeO}+m_{SiO_{2}}\right)}+\frac{M_{Fe}}{M_{C}}k_{dCD}\left(X_{C}-X_{C,EQ}\right)\;+2\frac{M_{Fe}}{M_{Si}}k_{dsil}\left(X_{Si}-X_{Si,EQ}\right)-\frac{2M_{Fe}O_{2,lance}}{M_{O2}}\;\;+K_{FeDRI}{\overset{˙}{m}}_{DRI}+0.945{\overset{˙}{m}}_{HM}$$

The rate of change of solid slag is function of the mass rate of lime, dolomite and the slag melting. The slag melting rate depends on the temperature difference between the liquid and the solid phase. The solid slag receives sensible heat and latent heat to change the phase into liquid (Equation (5)).

(5) $$\frac{dm_{ssl}}{dt}=-\frac{M_{Sl}K_{area5}K_{therm5}m_{ssl}\left(T_{lsc}-T_{ssc}\right)}{\left(\lambda_{ssl}+c_{p,sl}\left(T_{ssc}-T_{lsc}\right)\right)}\frac{T_{ssc}}{T_{lsc}}+{\overset{˙}{m}}_{lime}+{\overset{˙}{m}}_{dolmt}$$

The rate of change of liquid slag is equal to the melting rate of the solid slag as follows (Equation (6)).

(6) $$\frac{dm_{lsl}}{dt}=\frac{M_{Sl}K_{area5}K_{therm5}m_{ssl}\left(T_{lsc}-T_{ssc}\right)}{\left(\lambda_{ssl}+c_{p,sl}(T_{ssc}-T_{lsc})\right)}\frac{T_{ssc}}{T_{lsc}}$$

The CO mass rate in the gas phase is function of reaction of FeO with graphite injected into the slag $\left\{\frac{M_{CO}}{M_{C}}\frac{m_{FeO}k_{dCl}m_{C}}{\left(m_{lsl}+m_{FeO}+m_{SiO_{2}}\right)}\right\}$, extraction of gas through the off-gas duct $\left\{\frac{h_{d}u_{1}m_{CO}}{\left(k_{u}u_{2}+h_{d}\right)\left(m_{CO}+m_{CO_{2}}+m_{N_{2}}\right)}\right\}$, reaction of CO with graphite injected into the slag $\left\{\frac{M_{CO}C_{inj}}{M_{C}}\right\}$, decarburization of the steel bath $\left\{\frac{M_{CO}k_{dCD}\left(X_{C}-X_{C,EQ}\right)}{M_{C}}\right\}$, combustion of CO in leak-air $\left\{M_{CO}k_{Air1}k_{pr}P\right\}$, reaction CO with O2 lance $\left\{\frac{M_{CO}}{M_{C}}k_{dc1}\left(X_{C}-X_{C,EQ}\right)\;O_{2,\;lance}K_{O2CO}\right\}$. In total, the CO mass rate is formulated as follows (Equation (7)).

(7) $$\frac{dm_{CO}}{dt}=\frac{M_{CO}}{M_{C}}\frac{m_{FeO}k_{dCl}m_{C}}{\left(m_{lsl}+m_{FeO}+m_{SiO_{2}}\right)}\;\;-\frac{h_{d}u_{1}m_{CO}}{\left(k_{u}u_{2}+h_{d}\right)\left(m_{CO}+m_{CO_{2}}+m_{N_{2}}\right)}+\frac{M_{CO}C_{inj}}{M_{C}}\;\;+\frac{M_{CO}k_{dCD}\left(X_{C}-X_{C,EQ}\right)}{M_{C}}\;\;+M_{CO}k_{Air1}k_{pr}P+\frac{M_{CO}}{M_{C}}k_{dc1}\left(X_{C}-X_{C,EQ}\right)O_{2,lance}K_{O2CO}$$

The CO_2_ in the gas-phase depends on 3 mechanisms, i.e., the extraction of gas through the off-gas duct $\left\{\frac{h_{d}u_{1}m_{CO_{2}}}{\left(k_{u}u_{2}+h_{d}\right)\left(m_{CO}+m_{CO_{2}}+m_{N_{2}}\right)}\right\}$, the rate of CO combustion to CO${}_{2}\;\left\{2M_{CO_{2}}k_{Air1}k_{pr}P\right\}$, and combustion of CH_4_ into CO${}_{2}\;\left\{n_{CH4,in}M_{CO_{2}}\right\}$ (Equation (8)).

(8) $$\frac{dm_{CO_{2}}}{dt}=-\frac{h_{d}u_{1}m_{CO_{2}}}{\left(k_{u}u_{2}+h_{d}\right)\left(m_{CO}+m_{CO_{2}}+m_{N_{2}}\right)}\;\;-2M_{CO_{2}}k_{Air1}k_{pr}P+n_{CH4,in}M_{CO_{2}}$$

The N_2_ in the gas-phase is calculated from the extraction of gas through the off-gas duct, the N_2_ leaking in with the leak air (Equation (9)).

(9) $$\frac{dm_{N_{2}}}{dt}=-\frac{h_{d}u_{1}m_{N_{2}}}{\left(k_{u}u_{2}+h_{d}\right)\left(m_{CO}+m_{CO_{2}}+m_{N_{2}}\right)}-M_{N2}k_{Air1}k_{pr}P$$

The rate of change for C in the furnace is determined by the rate of the DRI, hot metal, graphite injection, carbon melting and reaction C with FeO as follows (Equation (10)).

(10) $$\frac{dm_{C}}{dt}=k_{C,DRI}{\overset{˙}{m}}_{DRI}+k_{C,HM}{\overset{˙}{m}}_{HM}+C_{inj}-C_{melt}-\frac{m_{FeO}k_{dCl}m_{C}}{\left(m_{lsl}+m_{FeO}+m_{SiO_{2}}\right)}$$

The rate of change for FeO in the slag is controlled by the rate of Fe oxidation by oxygen injection, the DRI input rate, the FeO reduction by carbon and silicon from the steel melt and the reduction of FeO by graphite (Equation (11)).

(11) $$\frac{dm_{FeO}}{dt}=\frac{2M_{FeO}}{M_{O2}}O_{2,lance}K_{O_{2},FeO}+{\overset{˙}{m}}_{DRI}K_{FeO,DRI}\;\;+\frac{2M_{FeO}}{M_{Si}}k_{dsil}\left(X_{Si}-X_{Si,Eq}\right)-\frac{M_{FeO}}{M_{C}}k_{dCD}\left(X_{C}-X_{C,Eq}\right)\;-\frac{m_{FeO}k_{gr}M_{Fe}C_{inj}}{M_{C}\left(m_{lsl}+m_{FeO}+m_{SiO_{2}}\right)}$$

The rate of change of dissolved carbon is determined by 4 factors. First, the rate of carbon in the steel melt is defined by $\frac{m_{C}T_{lsc}c_{p,lsc}}{\left(\lambda_{C}+c_{p,C}(T_{m}-T_{a})\right)}\frac{T_{a}}{T_{m}}$. Second, the reaction between C and O_2_ into CO with equation $k_{dc1}\left(X_{C}-X_{C,EQ}\right)\;O_{2,\;lance}K_{O_{2}CO}$. Third, reaction between C and O_2_ into CO_2_ with equation $k_{dc2}\left(X_{C}-X_{C,EQ}\right)\;O_{2,\;lance}K_{O_{2}CO_{2}}$. The rate of decarburization due to reaction with FeO in the slag is formulated with $k_{dcD}\left(X_{C}-X_{C,EQ}\right)$. The full equation is presented as follows (Equation (12)).

(12) $$\frac{dm_{C,diss}}{dt}=\frac{m_{C}T_{lsc}c_{p,lsc}}{\left(\lambda_{C}+c_{p,C}(T_{m}-T_{a})\right)}\frac{T_{a}}{T_{m}}-k_{dc1}\left(X_{C}-X_{C,EQ}\right)O_{2,lance}K_{O_{2}CO}\;\;-k_{dc2}\left(X_{C}-X_{C,EQ}\right)O_{2,lance}K_{O_{2}CO_{2}}-k_{dcD}\left(X_{C}-X_{C,EQ}\right)$$

The rate of change of SiO_2_ is determined by the difference fraction of Si in the steel melt and Si at equilibrium (Equation (13)). The SiO_2_ content in DRI input also contributed into the rate of change SiO_2_.

(13) $$\frac{dm_{SiO_{2}}}{dt}=\frac{M_{SiO_{2}}}{M_{Si}}k_{dsil}\left(X_{Si}-X_{Si,Eq}\right)+K_{SiO_{2},DRI}m_{DRI}$$

The rate of decrease of the silicon ($m_{Si}$) in the steel melt is formulated as the function of the difference content of Si in the steel melt and Si at equilibrium (Equation (14)).

(14) $$\frac{dm_{Si}}{dt}=-k_{dsil}\left(X_{Si}-X_{Si,Eq}\right)$$

Here are supporting equations for the differential equations above, i.e. enthalpy of reactions, heat transfer and sensible heats (Equation (15)-(27)).

(15) $${\overset{˙}{Q}}_{t}={\overset{˙}{Q}}_{1}+{\overset{˙}{Q}}_{2}+{\overset{˙}{Q}}_{3}+{\overset{˙}{Q}}_{4}+{\overset{˙}{Q}}_{5}+{\overset{˙}{Q}}_{6}+{\overset{˙}{Q}}_{7}+{\overset{˙}{Q}}_{8}+{\overset{˙}{Q}}_{9}+{\overset{˙}{Q}}_{10}+{\overset{˙}{Q}}_{11}+{\overset{˙}{Q}}_{12}$$

(16) $${\overset{˙}{Q}}_{1}=\left(\Delta H_{C-S}+\Delta H_{FeO}-\Delta H_{CO}\right)\frac{k_{dCD}}{M_{C}}\left(x_{C}-X_{cEQ}\right)$$

(17) $${\overset{˙}{Q}}_{2}=-\Delta H_{FeO}\frac{O_{2lance}}{M_{O_{2}}}$$

(18) $${\overset{˙}{Q}}_{3}=\left(\Delta H_{CO_{2}}-\Delta H_{CO}\right)2k_{AIR1}k_{pr}p$$

(19) $${\overset{˙}{Q}}_{4}=\left(\Delta H_{SiO_{2}}-\Delta H_{SiO2,s}-\Delta H_{Si,s}\right)\frac{k_{dsil}\left(X_{Si}-X_{Si,Eq}\right)}{M_{Si}}$$

(20) $${\overset{˙}{Q}}_{5}=\left(\frac{-O_{2,lance}}{M_{O2}}\right)\left(T_{lsc}-T_{O2}\right)c_{pO2}$$

(21) $${\overset{˙}{Q}}_{6}=k_{Air2}k_{pr}p\left(T_{lsc}-T_{air}\right)c_{pO2}$$

(22) $${\overset{˙}{Q}}_{7}=k_{Air2}k_{pr}p\left(T_{lsc}-T_{air}\right)c_{pN2}$$

(23) $${\overset{˙}{Q}}_{8}=\frac{-2\left(lime+dolmt\right)c_{pssl}}{M_{slag}}\left(Tssc-T_{slag}\right)$$

(24) $${\overset{˙}{Q}}_{9}=\frac{-\left(1-K_{FeOdri}\right)DRI}{M_{Fe}}\left(L_{ssc}+c_{pFe}(Tssc-T_{DRI})\right)$$

(25) $${\overset{˙}{Q}}_{10}=-K_{therm1}K_{area1}m_{ssc}\left(T_{lsc}-T_{ssc}\right)-K_{therm5}K_{area5}m_{ssl}\left(T_{lsc}-T_{ssc}\right)$$

(26) $${\overset{˙}{Q}}_{11}=\frac{m_{FeO}k_{gr}C_{inj}\left(\Delta H_{FeO}-\Delta H_{CO}\right)}{M_{C}\left(m_{lsl}+m_{FeO}+m_{SiO2}\right)}$$

(27) $${\overset{˙}{Q}}_{12}=-nCH_{4,in}(\left(\Delta H_{CO_{2}}+2\Delta H_{H_{2}O}-\Delta H_{CH_{4}}\right)\;+(c_{p,CO_{2}}+2c_{p,H_{2}O}-c_{p,CH_{4}}-2c_{p,O_{2}})\left(T_{gas}-298\right))$$

This investigation studied three cases with details of different compositions of metal charge, including HM 0% DRI 50% (case 1 which is the existing condition), HM 30% DRI 50% (case 2), and HM 50% DRI 30% (case 3). Hot metal from blast furnace operation contains high energy which could reduce the power in the arc furnace. However, the addition of hot metal could lead a high temperature on the dedusting system which potentially hazardous to the bag filter in the dedusting section. Hence, the integrative simulation between EAF and the dedusting system evaluated with several cases as presented in Table 2.

**Table 2:** Three scenarios of hot metal input for simulation in the EAF.

EAF input	Case 01 (Existing condition)	Case 02	Case 03
	HM 0%	HM 30%	HM 50%
	DRI 50%	DRI 50%	DRI 30%
	Scrap 50%	Scrap 25%	Scrap 25%
Input (ton)			
Hot Metal	0	42	70
DRI	70	70	42
Scrap	70	28	28

### 2.2 Model development in dedusting system

**Figure 2:**
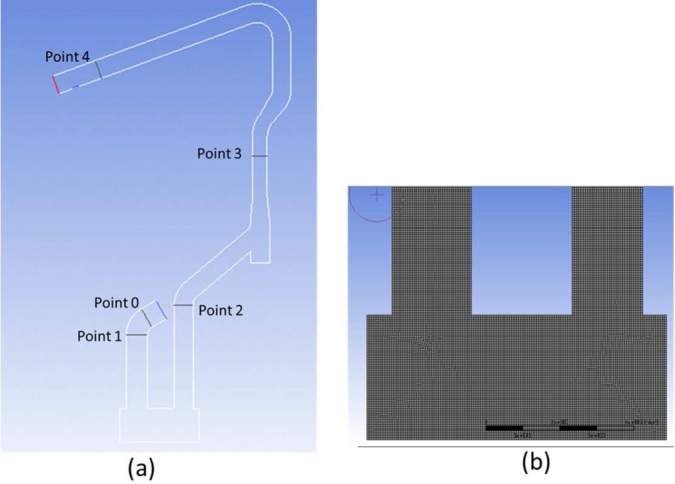
(a) Water-cooled duct (b) meshing generation in water-cooled duct area.

The results from EAF simulation, i.e., off-gas composition, temperature, etc., were used as an input for simulation in the water-cooled duct (WCD) of the dedusting system using CFD. Table 3 shows conditions simulation for WCD of a dedusting system for an existing condition. Fig. 2a shows the WCD part, and Fig. 2b presents meshing generation.

**Table 3:** Existing conditions for simulation water-cooled duct of dedusting.

Off gas parameter (units)	Value
Flowrate (kg/h)	51575
Temperature (K)	2073
Linear velocity (m/s)	36
Pressure outlet (Pa)	−6700
Temperature outlet (K)	848
Heat flux (kW/m^2^)	−160
Electrode diameter (mm)	610
Ambient temperature (K)	308
Dust flowrate (kg/h)	1463
Dust particle size (mm)	3

The Eulerian-Lagrangian method is used to model mass, energy and momentum balance between two phases (off-gas and dust) in the WCD (Equation (28)). The equation in this present work adapted from [17] and they are expressed in a partial differential equation.

(28) $$\frac{\partial \rho \Phi }{\partial t}+u_{i}\frac{\partial \rho \Phi }{\partial x_{i}}=\frac{\partial }{\partial x_{i}}\left(\Gamma_{\Phi }\frac{\partial \Phi }{\partial x_{i}}\right)+S_{\Phi }$$

Where the variable Φ can be velocity (u,v,w), concentration ($Y_{i}$), energy (Temperature, *T* or enthalpy, *H*), turbulent kinetic energy *k*, turbulent dissipation rate *ε* and other variables.

The turbulent model is used to found a method of evaluating the value of turbulent viscosity ($\mu_{t}$) [12]. There are two turbulent variables in the RNG-*kε* equation, that is turbulent kinetic energy (*k*) and turbulent dissipation energy (*ε*) (Equation (29)-(30)). The *k*-*ε* model is not recommended for vortex flows. In this case it is assumed that the fluid flowing in the WCD does not contain vortex.

(29) $$\;\rho \frac{\partial k}{\partial t}+\rho {\overset{¯}{u}}_{x}\frac{\partial k}{\partial x}+\rho {\overset{¯}{u}}_{y}\frac{\partial k}{\partial y}+\rho {\overset{¯}{u}}_{z}\frac{\partial k}{\partial z}=\frac{\partial }{\partial x}\left[\left(\mu +\frac{\mu_{t}}{\sigma_{k}}\right)\frac{\partial k}{\partial x}\right]+\frac{\partial }{\partial y}\left[\left(\mu +\frac{\mu_{t}}{\sigma_{k}}\right)\frac{\partial k}{\partial y}\right]+\frac{\partial }{\partial z}\left[\left(\mu +\frac{\mu_{t}}{\sigma_{k}}\right)\frac{\partial k}{\partial z}\right]\;+P_{k}-\rho \varepsilon$$

(30) $$\rho \frac{\partial \varepsilon }{\partial t}+\rho {\overset{¯}{u}}_{x}\frac{\partial \varepsilon }{\partial x}+\rho {\overset{¯}{u}}_{y}\frac{\partial \varepsilon }{\partial y}+\rho {\overset{¯}{u}}_{z}\frac{\partial \varepsilon }{\partial z}=\frac{\partial }{\partial x}\left[\left(\mu +\frac{\mu_{t}}{\sigma_{k}}\right)\frac{\partial \varepsilon }{\partial x}\right]+\frac{\partial }{\partial y}\left[\left(\mu +\frac{\mu_{t}}{\sigma_{k}}\right)\frac{\partial \varepsilon }{\partial y}\right]+\frac{\partial }{\partial z}\left[\left(\mu +\frac{\mu_{t}}{\sigma_{k}}\right)\frac{\partial \varepsilon }{\partial z}\right]\;+C_{\varepsilon 1}\frac{\varepsilon }{k}P_{k}-C_{\varepsilon 2}\rho \frac{\varepsilon^{2}}{k}$$

The rate of turbulent kinetic production $P_{k}$ is obtained from (Equation (31))

(31) $$P_{k}=2\mu_{t}\left[{\left(\frac{\partial {\bar{u}}_{x}}{\partial x}\right)}^{2}+{\left(\frac{\partial {\bar{u}}_{y}}{\partial y}\right)}^{2}+{\left(\frac{\partial {\bar{u}}_{z}}{\partial z}\right)}^{2}\right]+\mu_{t}{\left(\frac{\partial {\bar{u}}_{x}}{\partial y}+\frac{\partial {\bar{u}}_{y}}{\partial x}\right)}^{2}\;\;+\mu_{t}{\left(\frac{\partial {\bar{u}}_{x}}{\partial z}+\frac{\partial {\bar{u}}_{z}}{\partial x}\right)}^{2}+\mu_{t}{\left(\frac{\partial {\bar{u}}_{z}}{\partial y}+\frac{\partial {\bar{u}}_{y}}{\partial z}\right)}^{2}$$

Turbulent viscosity ($\mu_{t}$) for the RNG-*kε* turbulent model derived from the boussinesq hypothesis (Equation (32)) [18].

(32) $$\mu_{t}=\mu_{t0}f\left(\alpha_{S},\Omega ,\frac{k}{\varepsilon }\right)$$

Where $\mu_{t0}$ is the standard turbulent viscosity calculated without vortex, *μ*_t0_ = $C_{\mu }\rho k^{2}/\varepsilon$. $\alpha_{s}$ is a vortex flow factor whose value is assumed to differ depending on the magnitude of the vortex in the flow. Ω is a vortex characteristic value. Some constants that need to be sought in solving the turbulent model are $C_{\varepsilon 2}$ and *λ*. The value of the constants of the RNG-*kε* model is $C_{\varepsilon 1}=1.42$; ${\overset{˜}{C}}_{\varepsilon 1}=1.68$; Cμ=0.085; $\sigma_{k}=0.72$; $\sigma_{s}=0.72$; β=0.012; and $\lambda_{0}=4.38$ [19].

The number of elements was more than 50,000. Amongst various mesh sizes and shapes that have been investigated were quadrilateral dan trilateral, with mesh sizes of 60 mm and up to 50,000 elements used. To assess the quality of the tested meshed there are several parameters selected, including aspect ratio, skewness, and orthogonality. The aspect ratio is a measure of the stretching of a cell. It is computed as the ratio of the maximum value to the minimum value of any of the following distances: the distances between the cell centroid and face centroids, and the distances between the cell centroid and nodes. The values are scaled, so that an aspect ratio of 1 corresponds to a perfectly regular element, while an aspect ratio of 0 indicates that the element has zero volume. In this present work, the maximum aspect ratio is 1.8779 and the minimum is 1 (90% mesh face).

The skewness cells should be as low as possible, the greater the skewness could result in solver convergences issues [20]. In all cases in this work it is normalized so that 0 is ideal and 1 is the worst possible. In this present work, more than 90% mesh has skewness value 0, and maximum skewness value is 0.621. The orthogonality quality depends on cell type, the worst cells will have an orthogonal quality close to 0 while the best cells will have an orthogonal quality close to 1. In this present work, the maximum value of orthoganolity value is 1 (more than 98% mesh) and the minimum is 0.76.

## 3 Results and discussion

### 3.1 Model validation

Table 4 presents the feed composition of EAF for each material in the furnace. Carbon content in both DRI and hot metal is ca. 4%, which is considerably high carbon in steelmaking processing. On the other hand, according to the EAF plant data, the scrap content is only 0.15%.

**Table 4:** The feed composition of EAF.

Input	Fe (%)	FeO (%)	C (%)	Si (%)	Traces (%)
Hot heel	99.00	0	0.06	0	0.94
Scrap	95.00	0	0.15	0	4.85
DRI	81.00	8.70	3.84	0	6.46
Hot Metal	94.50	0	4.00	1.00	0.50

The mathematics model (Equation (1) – (27)) was solved using MATLAB® with the ode15s iteration to solve rigid differential equations. Thus, the simulation results were compared with a real furnace data plant to validate the developed model for two actual operations, i.e., 1 and 2 times charging scrap. The scrap was charging into the EAF only one times at the beginning (0 min) for the 1 times charging scrap. For the 2 times charging scrap, the scrap was introducing into the EAF 2 times at 0 min and 20 min. Three parameters, i.e., liquid temperature, the mass of produced liquid steel, and carbon content in liquid steel, of actual data were compared with the simulation by calculating the error ($abs\;(\frac{Actual\;-Simulation}{Actual})$). For the first case, the error between simulation results and data plant of liquid temperature, the mass of produced liquid steel, and carbon content in liquid steel were 0.06%, 2.8%, and 0.074%, respectively. For the second case, the error of liquid temperature, the mass of produced liquid steel, and carbon content in liquid steel were 0.25%, 1.7%, and 4%, respectively. The error suggested that the model is valid for further simulation purposes.

### 3.2 Simulation results of an existing condition

**Figure 3:**
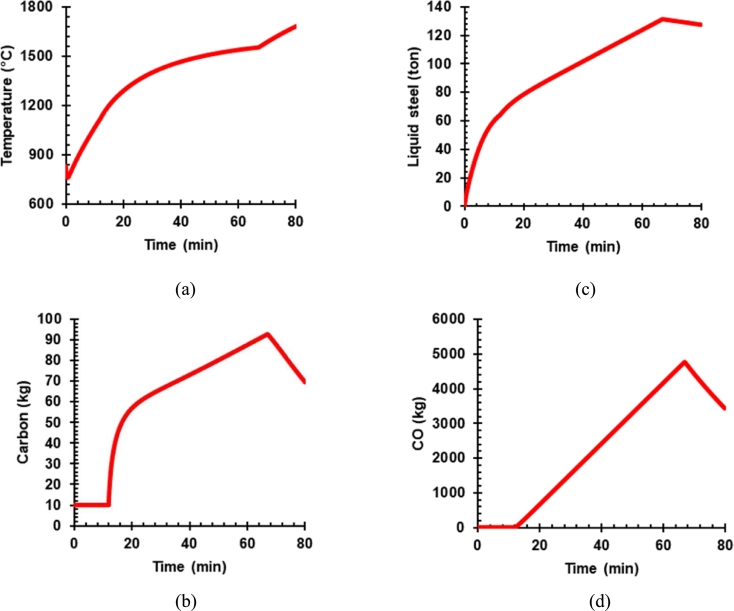
Simulation result of existing EAF operation (a) temperature of the liquid steel (b) the mass of dissolved carbon (c) the mass of liquid steel (d) the mass of carbon monoxide.

Fig. 3 shows the simulation results of EAF with existing condition operation in which initially liquid steel temperature in the furnace was represented by hot heel mixed with scrap, lime, and dolomite. The liquid steel temperature dropped as the heat transferred into the scrap for increasing temperature and melting the scrap.

The liquid steel temperature gradually increased as the electric arc power turned on (Fig. 3a). At the point when DRI is charging into the furnace with a high energy supply for iron melting indicated in the graphic with a slower increasing liquid steel temperature rate.

Fig. 3b presents carbon evolution which is started at minute twelve when graphite is introduced into the reactor. It is a surge increasing at the beginning of carbon in a graphite form charging, while the carbon accumulation rate decreases until graphite injection turns off after 67 minutes of operation. The graphite accumulation was reduced when the consumption of decarburization with iron oxide and oxygen no longer existed. The carbon accumulation also counts from the DRI process.

Fig. 3c depicts the mass of liquid steel produced over time. For the first 12 minutes, liquid steel was contributed solely from scrap melting. DRI gradually charged into the furnace from 12 to 67 minutes, which became the main contributor to liquid steel production unless the hot metal proportion was charging larger than DRI.

In addition, Fig. 3d shows carbon monoxide formation during furnace operation. Carbon monoxide was produced from the reaction between FeO and Carbon (C). The Carbon Monoxide (CO) compound stopped forming as the DRI and carbon riser off after 67 minutes. Hence, some CO components have been partly oxidized into CO_2_ and released, increasing the furnace temperature.

### 3.3 Effect of hot metal charging on the temperature of liquid steel

**Figure 4:**
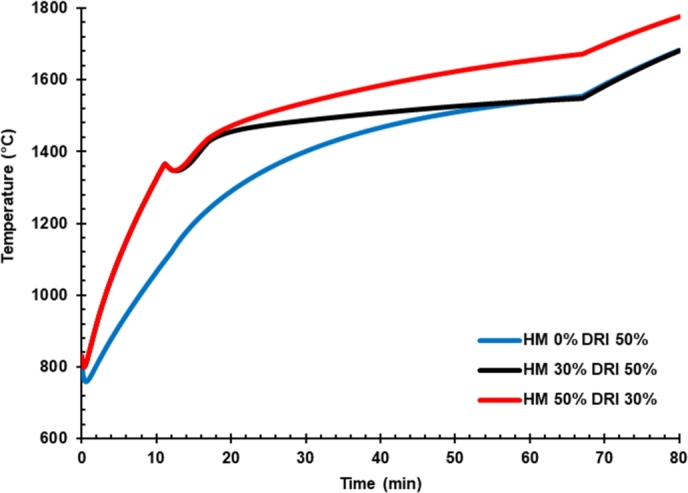
Effect of hot metal charging on the temperature of liquid iron.

Fig. 4 shows the temperature of liquid steel over the time of furnace operation for cases 1, 2, and 3. The simulation was performed with constant total electrical arc power at 87.4 MWh. The effect of hot metal charging on liquid iron temperature is seen in case 3, with 50% of hot metal charging being higher than without hot metal charging during the whole batch processing. This event was caused by scrap charging that decreased from 50% to 20% while hot metal was charged into the furnace. Therefore, the energy required for melting the scrap reduces significantly. The heat from electric arc power and hot metal is partially consumed for sensible heat to increase the liquid iron temperature.

Increasing hot metal charging from 30% to 50% by reducing DRI charging from 50% to 30% favored high-temperature liquid iron. Hot metal caries high energy compared to DRI and no energy requirement for phase changing. DRI, which contained high FeO ca. 8%, was reduced into Fe via decarburization.

The reaction of the process is endothermic. Hence it is required energy supplied from the liquid iron; the most demanding energy was from the energy required to melt the solid iron in DRI. This phenomenon explains why the temperature is lower in high DRI rate loading. The rate of decarburization reaction was significantly affected by carbon content in the DRI and temperature, as reported elsewhere [21].

### 3.4 Effect of hot metal charging on the mass of liquid steel

**Figure 5:**
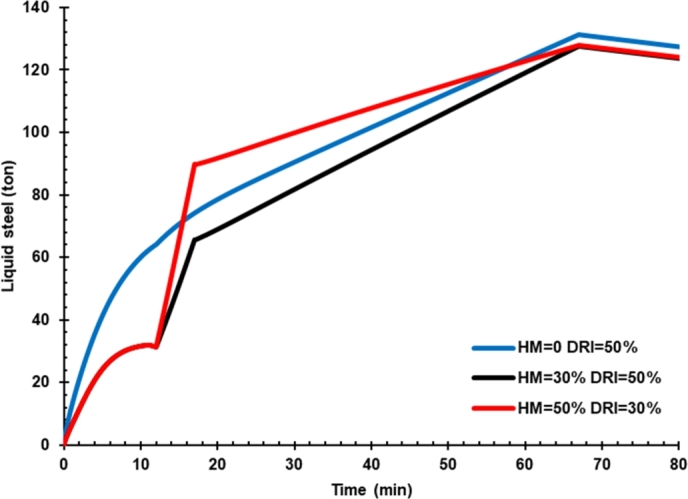
Effect of hot metal charging on the mass of liquid iron.

Fig. 5 presents the effect of hot metal charging on the mass of liquid steel. Despite high carbon content (ca. 4%) in hot metal-assisted decarburization of FeO to Fe in the DRI units, hot metal charging on the production rate of liquid steel shows that hot metal with lower Fe content (ca. 94.5%) caused the rate of liquid steel production slightly decrease.

Typically, hot metal composition impurities up to 6% produced from blast furnace operation are C, Si, Mn, P, S, V, and Ti [22]. With the high Fe content of hot metal, the liquid steel produced from EAF would be higher. Iron content in hot metal depends on the ore raw materials, coke properties, and operating conditions of the blast furnace.

### 3.5 Effect of hot metal charging on carbon in liquid steel and CO formation

**Figure 6:**
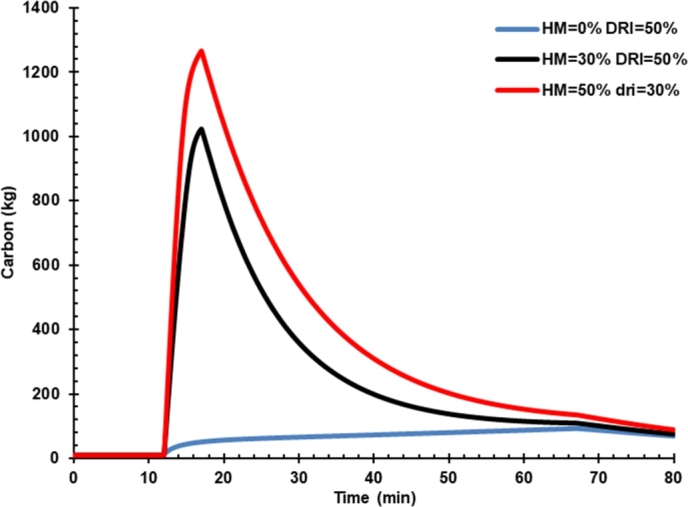
Effect of hot metal charging on dissolved carbon in liquid iron.

**Figure 7:**
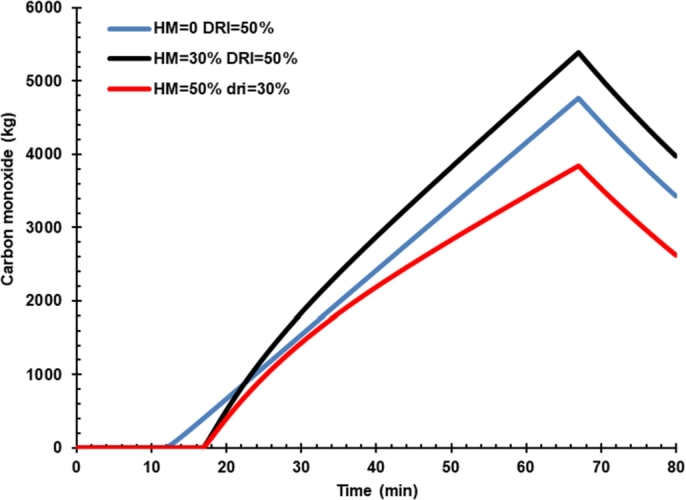
Effect of hot metal charging on carbon monoxide.

Fig. 6 shows the effect of hot metal charging on dissolved carbon in liquid iron. Carbon content in case 3 was higher than hot metal charging case 2 because the hot metal has a higher carbon than DRI, as presented in Table 3. Thus, the oxygen flow rate controls the carbon content in liquid steel. The carbon produced will react with the oxygen to form CO molecules that carry the heat to be released (Fig. 7). CO in off-gas must be reduced from the EAF by increasing the oxygen content in the arc furnace so that the CO oxidation process to CO_2_ can be performed inside the furnace. The presence of additional energy from the conversion of C to CO and CO_2_ must be balanced with decreasing the arc power consumption during the process. Thus, the presence of C in hot metal should not be a problem and even can increase the energy and reduce electrical energy consumed instead. However, post-combustion CO in the dedusting area would be burdening the cooling system. Hot metal charging was reported to decrease carbon emission as the power was reduced significantly, and carbon emission could be further reduced by gas waste heat utilization [4].

The effect of hot metal charging on EAF performance is summarized in Table 5. An increase in hot metal by up to 50% caused liquid steel production flow rate to decrease compared with no hot metal charging. In addition, the slag content also increases, and the FeO content in the slag decreases.

**Table 5:** Effect of hot metal charging on EAF products and power consumption.

EAF input and products	Case 01	Case 02	Case 03
	HM 0%	HM 30%	HM 50%
	DRI 50%	DRI 50%	DRI 30%
**Input (ton)**			
Hot Metal	0	42	70
DRI	70	70	42
Scrap	70	28	28
**Product (units)**			
Liquid steel (ton)	127	123.7	124
Carbon (%)	0.050	0.057	0.066
Slag (ton)	17.0	20.7	22.6
**Power Consumption (MWh)**	87.4	81.2	72.9

Hot metal charging reduced arc power consumption from 87.4 MWh to 81.2 and 72.9 MWh for cases 2 and 3. However, the carbon content in liquid steel increased from 0.05% to 0.057% and 0.066% for cases 2 and 3, respectively. Hence, it is necessary to increase the oxygen rate to reduce carbon content when charging hot metal into EAF.

### 3.6 Effect of oxygen flow rate on the temperature of liquid steel

Hot metal charging generally has high carbon content and causing to burdening the decarburization process [8]. One solution that can overcome this issue is increasing the oxygen rate through an oxygen lance. Table 6 presents oxygen flow rate variation in case 2, i.e., HM 30% DRI 50%. Oxygen injection into liquid steel led to various consequences such as high temperature, slag, and product quality improvement. The simulations of the smelting process have been carried out on fixed hot metal conditions at 30% composition and observed at the various O_2_ lance rate at the consumption during the smelting process of 1800, 3600, and 5400 Nm^3^. Simulations were performed to study the effect of O_2_ rate on the temperature of liquid steel, the production rate of liquid steel, the carbon content in liquid steel, slag, and CO content in the off-gas.

**Table 6:** Oxygen flow rate variation in case 2.

*O*_2_ lance number	Oxygen rate (Nm^2^/h)
01	1,800
02	3,600
03	5,400

**Figure 8:**
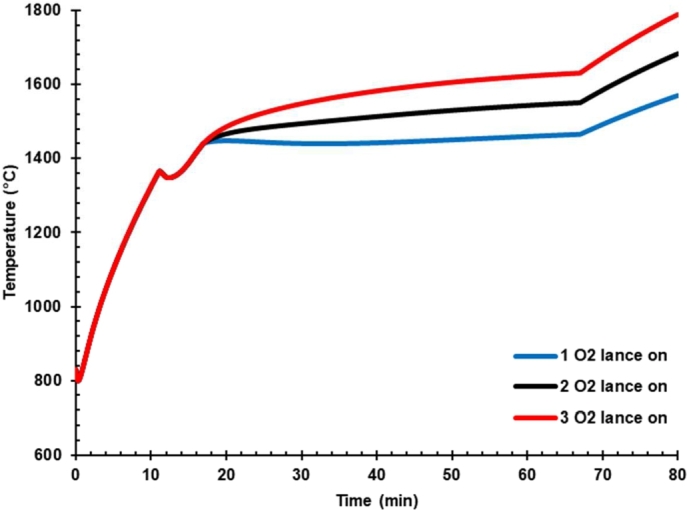
Effect of oxygen lance on liquid iron temperature.

Fig. 8 presents the effect of oxygen lance on liquid iron temperature. It was reported that blowing oxygen into hot metal improves the temperature of liquid iron [6]. It shows that the oxygen lance rate increases the liquid steel temperature. The heat is generated due to the oxidation process of carbon. Fig. 8 shows that the temperature at the oxygen lance rate of 5400 Nm^3^ increase the temperature up to 1800 °C.

The high temperature of EAF is proportionally positive with the off-gas temperature. Since the high temperature of the off-gas would overburden the cooling system, reducing arc power consumption to below the reactor and off-gas temperature would be necessary.

### 3.7 Effect of oxygen flow rate on carbon in liquid steel and CO formation

**Figure 9:**
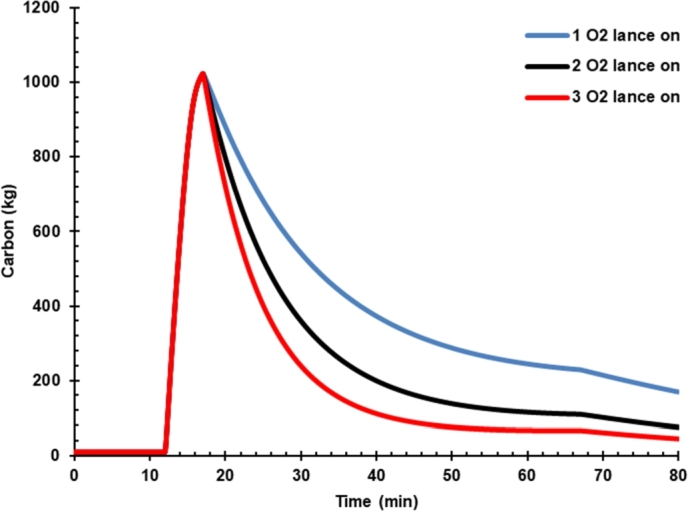
Effect of oxygen lance on dissolved carbon.

Fig. 9 presents the effect of oxygen lance on dissolved carbon in liquid steel. It is seen that dissolved carbon reduced significantly from 0.12% to 0.056% and 0.035% after increasing the oxygen rate from 1800 Nm^3^/h to 3600 Nm^3^/h and 5400 Nm^3^/h, respectively. Increasing the oxygen rate will effectively reduce carbon dissolved in liquid steel as the carbon oxidizes into CO or CO_2_.

**Figure 10:**
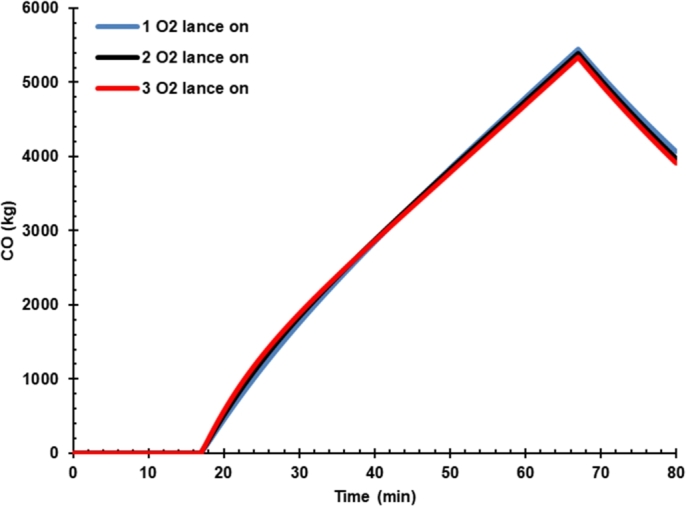
Effect of oxygen lance on the mass of carbon monoxide.

Fig. 10 shows the effect of oxygen lance on the mass of carbon monoxide. The effect of oxygen rate is not that much significant to carbon monoxide. This phenomenon is because oxygen is designed for oxidizing dissolved carbon.

Furthermore, the CO in the gas phase is not that much affected by the variation of oxygen lance rate unless oxygen lance is designed not only for oxidizing dissolved carbon but also for post-combustion CO in the EAF.

By increasing the O_2_ rate, the dissolved carbon in liquid steel significantly decreased (Table 7). Thus, the O_2_ rate must be optimized to meet the minimum requirement of dissolved carbon in liquid iron.

**Table 7:** Effect of oxygen flow rate on the mass of liquid steel and %-C.

Product	One lance	Two lance	Three lance
Liquid steel (ton)	129	124	119
Carbon (%)	0.12	0.056	0.035

### 3.8 Effect of hot metal charging on dedusting duct area

**Figure 11:**
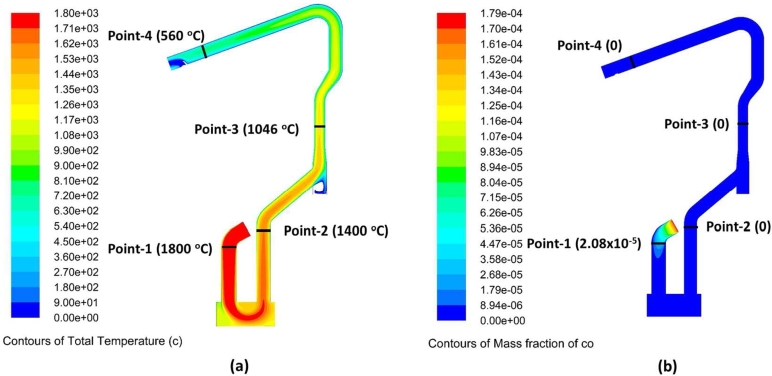
(a) Temperature contour of water-cooled duct area of the dedusting system without hot metal charging (b) contour of CO mass fraction.

The initial simulation stage with CFD in the dedusting area without hot metal charging is by determining the operating parameters. The mass flow rate of off-gas entering the WCD area is 50 ton/h, and infiltrated air was considered. The off-gas temperature from the arc reactor is 1800 °C. The high off-gas temperature was reduced to 575 °C at the exit of WCD. Fig. 11a presents the temperature contour along the WCD area. It can be seen in Fig. 11a that area before the dropbox has a high temperature of 1800 °C (point 1) and exit the drop box at 1400 °C (point 2). The cooling water flow rate must be able to control a low target of off-gas temperature of 560 °C at the exit point of WCD (point-4). Besides cooling water, the ambient air was also injected at point 3 to assist the cooling process of the off-gas.

The high CO content in the dedusting area before the dropbox caused a post-combustion CO to increase the temperature (Fig. 11 b). The phenomena agreed with Tang et al., that reported CFD simulation of dedusting system area showed the additional air and CH_4_ injection will improve CO conversion. However, it increases duct temperature [23]. The heat transfer rate can be increased by increasing the cooling water rate and cooling air mixed at point 3.

**Figure 12:**
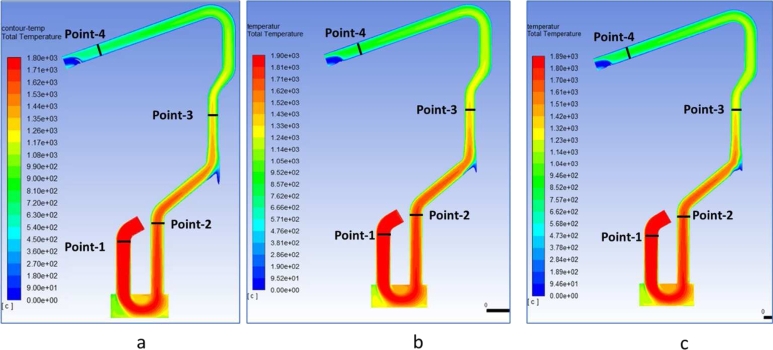
Comparison of temperature contour of water-cooled duct area (a) HM 0% DRI 50% (b) HM 30% DRI 50% (c) HM 50% DRI 30%.

Fig. 12a-c. shows the temperature contour of the water-cooled duct area for the case with and without hot metal charging. CFD simulations with hot metal for the dedusting area have been carried out at 30% and 50% Hot metal conditions. The simulation conditions in the EAF show that the temperature at the EAF off-gas with 50% hot metal is higher than that of 30% hot metal. However, the CO content at the EAF output in hot metal conditions is 30% higher than 50% (Fig. 6). The presence of CO in the off-gas increased the off-gas temperature due to post-combustion of CO.

**Figure 13:**
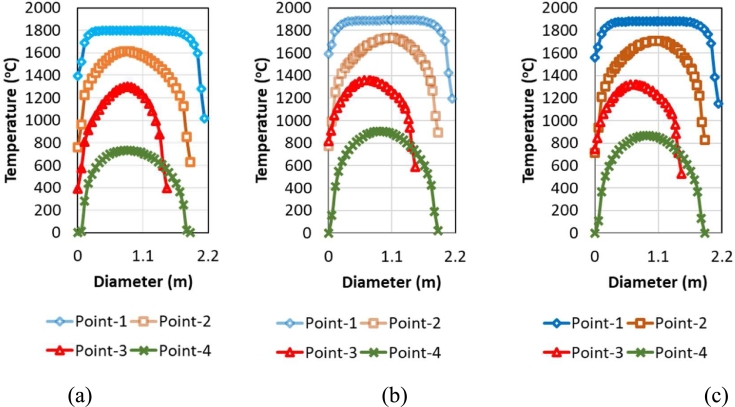
Comparison of temperature gas profile of water cooled duct area (a) HM 0% DRI 50% (b) HM 30% DRI 50% (c) HM 50% DRI 30%.

Fig. 13a-c depict the highest temperature point is ca.1900 °C. This temperature is higher than the off-gas temperature without hot metal, so the presence of hot metal increases the off-gas temperature along with the dedusting system. Hot metal charging increased the temperature of the dedusting duct because of the increase of flue gas temperature and post-combustion CO with infiltrated air in the WCD area. Heat recovery for steam generation is potentially applied to benefit from high temperature and cool the off-gas in the dedusting area [20].

**Figure 14:**
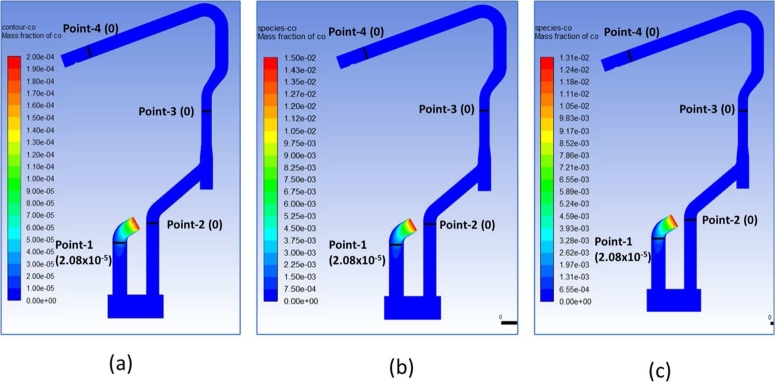
Comparison of CO mass fraction contour of water-cooled duct area (a) HM 0% DRI 50% (b) HM 30% DRI 50% (c) HM 50% DRI 30%.

Fig. 14a-c presents comparison of CO mass fraction contour of water-cooled duct area (a) HM 0% DRI 50% (b) HM 30% DRI 50% (c) HM 50% DRI 30%. All case studies showed that CO was completely oxidized before the drop-box area.

### 3.9 Post-combustion CO in the arc furnace

Post-combustion CO in the EAF instead of post-combustion CO in the dedusting area potentially reduces the electricity consumption of EAF. Oxygen lance for post-combustion CO in the arc furnace is designed with additional nozzles above the lance tip [21]. The reported design allows post-combustion CO to occur in the arc furnace. Table 8 presents simulation results for post-combustion CO in the arc furnace. A combination of hot metal charging of 30% and arc furnace power of 87.4 MWh exhibits such a high temperature of liquid steel at 1898 °C. The temperature of liquid steel increased, ca.1993 °C, while hot metal charging increased to 50%.

**Table 8:** Electric arc power reduction in case of hot metal charging with post-combustion CO.

Time (min)	HM 30%	HM 30%	HM 50%	HM 50%
	(High power)	(Low power)	(High power)	(Low power)
0 – 12	10.2	10.2	10.2	10.2
12 – 17	5.8	5.8	5.8	5.8
17 – 67	58.3	37.5	58.3	30.8
67 – 80	13.0	13.0	13.0	13.0
Total power (MWh)	87.4	66.5	87.4	59.9
T_end_ (°C)	1898	1628	1993	1642

**Table:** 

Terms	Notes	Unit
*m*_*ssc*_	Mass of solid scrap	kg
*m*_*ssl*_	Mass of solid slag	kg
*m*_*lsc*_	Mass of liquid scrap	kg
*m*_*lsl*_	Mass of liquid slag	kg
*m*_*C*_	Mass of C	kg
*m*_*C*,*diss*_	Mass of C dissolved in liquid steel	kg
*m*_*CO*2_	Mass of CO_2_	kg
*m*_*FeO*_	Mass of FeO	kg
*m*_*N*2_	Mass of N_2_	kg
*m*_*Si*_	Mass of Si	kg
*m*_*SiO*2_	Mass of SiO_2_	kg
${\overset{˙}{m}}_{lime}$	The mass rate of lime	kg/s
${\overset{˙}{m}}_{DRI}$	The mass rate of DRI	kg/s
${\overset{˙}{m}}_{dolmt}$	Mass rate of dolomite	kg/s
${\overset{˙}{m}}_{HM}$	The mass rate of hot metal	kg/s
*c*_*p*,*Fe*_	Heat capacity of Fe	kJ/(mol.K)
*c*_*p*,*FeO*_	Heat capacity of FeO	kJ/(mol.K)
*c*_*p*,*ssc*_	Heat capacity of solid scrap	kJ/(mol.K)
*c*_*p*,*ssl*_	Heat capacity of solid slag	kJ/(mol.K)
*c*_*p*,*lsl*_	Heat capacity of liquid slag	kJ/(mol.K)
*K*_*FeDRI*_	Mol fraction of Fe in DRI	−
*k*_*Fe*_	Heat transfer coefficient of iron	kW/(m^2^.K)
*k*_*ssl*_	Heat transfer coefficient of slag	kW/(m^2^.K)
*K*_*area*1_	Area constant for scrap	m^2^/kg
*K*_*therm*1_	Heat transfer coefficient of scrap	kW/(m^2^.K)
*K*_*area*5_	Area constant for slag	m^2^/kg
*K*_*therm*5_	Heat transfer coefficient of slag	kW/(m^2^.K)
*M*_*C*_	The molar mass of carbon	kg/mol
*M*_*Fe*_	The molar mass of iron	kg/mol
*M*_*Si*_	The molar mass of silicon	kg/mol
*M*_*sl*_	The molar mass of slag	Kg/mol
*P*_*arc*_	Power arc furnace	kW
${\overset{˙}{Q}}_{1}$	The heat of reaction C+FeO → Fe+CO	kW
${\overset{˙}{Q}}_{2}$	The heat of reaction Fe+1/2O_2_ → FeO	kW
${\overset{˙}{Q}}_{3}$	The heat of reaction CO+1/2O_2_ → CO_2_	kW
${\overset{˙}{Q}}_{4}$	Heat of reaction Si+FeO→Fe+SiO_2_	kW
${\overset{˙}{Q}}_{5}$	Heat loss to oxygen stream	kW
${\overset{˙}{Q}}_{6}$	Heat loss to oxygen leak air-stream	kW
${\overset{˙}{Q}}_{7}$	Heat loss to nitrogen leak air-stream	kW
${\overset{˙}{Q}}_{8}$	Heat loss to slag stream	kW
${\overset{˙}{Q}}_{9}$	Heat loss to DRI stream	kW
${\overset{˙}{Q}}_{10}$	Heat loss to solid scrap and slag	kW
${\overset{˙}{Q}}_{11}$	The heat of reaction due to C injection	kW
${\overset{˙}{Q}}_{12}$	The heat of reaction CH_4_ + *O*_2_ → CO_2_ + *H*_2_O	kW
*α*_*ssc*_	Area of heat transfer solid scrap	m^2^/kg
*α*_*ssl*_	Area of heat transfer slag	m^2^/kg
*T*_*lsc*_	The temperature of liquid scrap	K
*T*_*lsl*_	The temperature of liquid slag	K
*T*_*ssc*_	The temperature of solid scrap	K
*T*_*ssl*_	The temperature of solid slag	L
*T*_*m*_	Melting temperature of the iron	K
t	Time	s
*X*_*C*_	Mol fraction of C in the steel	
*X*_*Si*_	Mol fraction of Si in the steel	−
*X*_*C*,*EQ*_	Mol fraction of C equilibrium in the steel	−
*X*_*Si*,*Eq*_	Mol fraction of Si Equilibrium in the steel	−
*λ*_*ssc*_	Latent heat of scrap	kJ/mol
*λ*_*ssl*_	Latent heat of slag	kJ/mol
*ρ*	Fluid density	kg/m^3^
k	Specific turbulent kinetic energy	m^2^/s^2^
*ε*	Dissipation rate of turbulent kinetic energy	m^2^/s^3^
$\bar{u_{i}}$	Mean velocity vector	m/s

On the other hand, the temperature of liquid steel can be down to 1600-1650 °C by reducing electric arc furnace power from 87.4 MWh to 66.5 and 59.9 MWh in HM 30% and HM 50%, respectively, the liquid steel was maintained at a temperature range of 1600-1650 °C. A sophisticated model was also developed using CFD for CO post-combustion without hot metal. Sponge iron charging showed that the energy potency of the un-combusted CO in the burner and burner + lancing modes were 1.19 MW and 3.13 MW, respectively [22]. The potency of high formation CO from EAF operation could be tapped by post-combustion CO using an oxygen lance in the arc furnace.

## 4 Conclusions

Dynamic model and simulation of hot metal charging with cold DRI and scrap in EAF using MATLAB and CFD simulation in water-cooled duct of dedusting system have been performed. Three cases for the EAF with various hot metal (HM), sponge iron, and scrap iron charging compositions and four cases for the EAF with post-combustion CO have been successfully developed. The electric arc power consumption can be reduced down to 72.9 MWh from 87.4 MWh while at the same time increasing the HM charging temperature at the endpoint of the duct dedusting system up to 900 °C from 540 °C. The recycled energy can be potentially extracted from the dedusting system. Post-combustion CO in the arc furnace simulation shows that EAF power can be reduced from 87.4 MWh to 66.5 MWh and 59.9 MWh in case 2 and case 3, respectively. Hot metal charging combined with post-combustion CO in the EAF proves that the particular technique can be implied for better energy-efficient operation in EAF. The technique of integrating the conventional mathematics modeling with the CFD simulation could be further applied in other fields which has limitation while using the complicated CFD in some parts.

## Declarations

### Author contribution statement

Anton Irawan: Conceived and designed the experiments; Wrote the paper. Teguh Kurniawan: Performed the experiments; Wrote the paper. Hafid Alwan: Performed the experiments; Analyzed and interpreted the data. Zaenal Arifin Muslim, Hidayathul Akhmal, Mochamad Adha Firdaus: Analyzed and interpreted the data; Contributed reagents, materials, analysis tools or data. Yazid Bindar: Conceived and designed the experiments; Analyzed and interpreted the data

### Funding statement

This research did not receive any specific grant from funding agencies in the public, commercial, or not-for-profit sectors.

### Data availability statement

Data included in article/supp. material/referenced in article.

### Declaration of interests statement

The authors declare no conflict of interest.

### Additional information

No additional information is available for this paper.
